# Perineal stress as a predictor of performing episiotomy in primiparous women: a prospective observational study

**DOI:** 10.1186/s12884-022-05075-2

**Published:** 2022-10-26

**Authors:** Binbin Xu, Qi Luo, Rongrong Wu, Ying Lu, Hongjun Ying, Yanan Xu, Zhaie Lu

**Affiliations:** Ningbo Women & Children’s Hospital, NO.339, Liuting Street, Haishu District, 315012 Ningbo City, Zhejiang Province China

**Keywords:** Vaginal delivery, Perineal stress, Episiotomy, Midwife

## Abstract

**Background:**

Episiotomy is a surgical solution to relieve perineal stress, resulting in an easily repairable incision, in comparison to the risks of serious vaginal trauma during delivery. The midwife typically adopts such a clinical decision, on experience and subjective judgment. However, the association between perineal stress and episiotomy is poorly characterized. Our aim was to identify a threshold value for perineal stress leading to episiotomy, which eventually may be employed as a clinical tool for assessing whether an episiotomy is required or not.

**Methods:**

In total, 245 nulliparous women were investigated for perineal stress during non-instrumental vaginal delivery in Ningbo Women & Children’s Hospital. During the second stage of labor, a flexible membrane stress sensor was placed between the fetal head and perineal wall above the anal fissure. Once the entire fetal head pressed against the sensor, real-time perineal stress was measured, and the peak value was recorded. Cases were divided into non-episiotomy group (n = 173) and episiotomy group (n = 72). The correlations between perineal stress and episiotomy was assessed through logistic regression with adjustment for maternal age, estimated birthweight, duration of second stage of labor, maternal body mass index, and presence of analgesia. Midwives were blinded to all stress measurement values. The predictive value of perineal stress on performing episiotomy was evaluated, together with the ideal cut-off perineal stress value for performing episiotomy. A ROC analysis was also performed.

**Results:**

The episiotomy group had significantly higher levels of perineal stress in comparison to the non-episiotomy group (140.50 ± 16.03 N versus 118.37 ± 19.21 N, p < 0.01). The episiotomy group was linked to significantly higher perineal stress in comparison to the non-episiotomy group (140.50 ± 16.03 VS 118.37 ± 19.21 N, p < 0.01). ROC analysis between perineal stress and episiotomy revealed a high area under the curve (AUC 0.81, 95% CI 0.75–0.86) and a cut-off value for perineal stress of 124.49 N was identified for episiotomy decision.

**Conclusion:**

The level of perineal stress was an independent predictor of performing episiotomy in nulliparous women during non-instrumental vaginal delivery. Perineal stress exceeding 124.49 N was identified as the cut-off prompting midwives to perform episiotomy.

## Introduction

Episiotomy is the most prevalent surgical procedure performed in obstetrics [1]. Typically, this is performed when the perineum is identified as being taut. Selective episiotomies can occasionally circumvent soft-tissue ruptures during childbirth [2], with several studies demonstrating prophylaxis against obstetric anal sphincter injuries (OASIS) [3]. Selective episiotomy is better than routine episiotomy in non-instrumental vaginal birth [4], while it is likely more protective if performed routinely in instrumental vaginal birth [5]. However, if episiotomy is routinely performed in all vaginal deliveries, this could lead to excessive perineal injuries, postpartum pain, infection and other consequences [6]. Indications for episiotomy vary, based upon each individual nation’s professional practice. Episiotomy rates range between 20% and 70% in China [7]. In France, the episiotomy rate in 2016 was 34.9% in primiparous women [8]. An optimal episiotomy rate also requires balancing OASIS risk assessment against excess morbidity caused by the procedure, such as wound-healing complications [9]. There is no consensus regarding standard requirements for episiotomy, making subjective judgment of the attending midwife typically the sole factor in the clinical decision to perform an episiotomy [10].

Through the development of stress-sensing technology, dynamic monitoring of multiple body tissues can be successfully enabled. However, knowledge is scarce regarding perineal stress monitoring and its potential contribution to vaginal delivery.

This study aimed to address this knowledge gap by measuring perineal stress in nulliparous women during vaginal delivery and evaluating its effect on the clinical decision of episiotomy. This study hypothesized that elevated perineal stress is associated with the attending midwife’s decision on performing episiotomy, together with defining a cut-off value for perineal stress in order to prompt the midwife to perform an episiotomy.

## Materials and methods

### Study design and participants

**Fig. 1:**
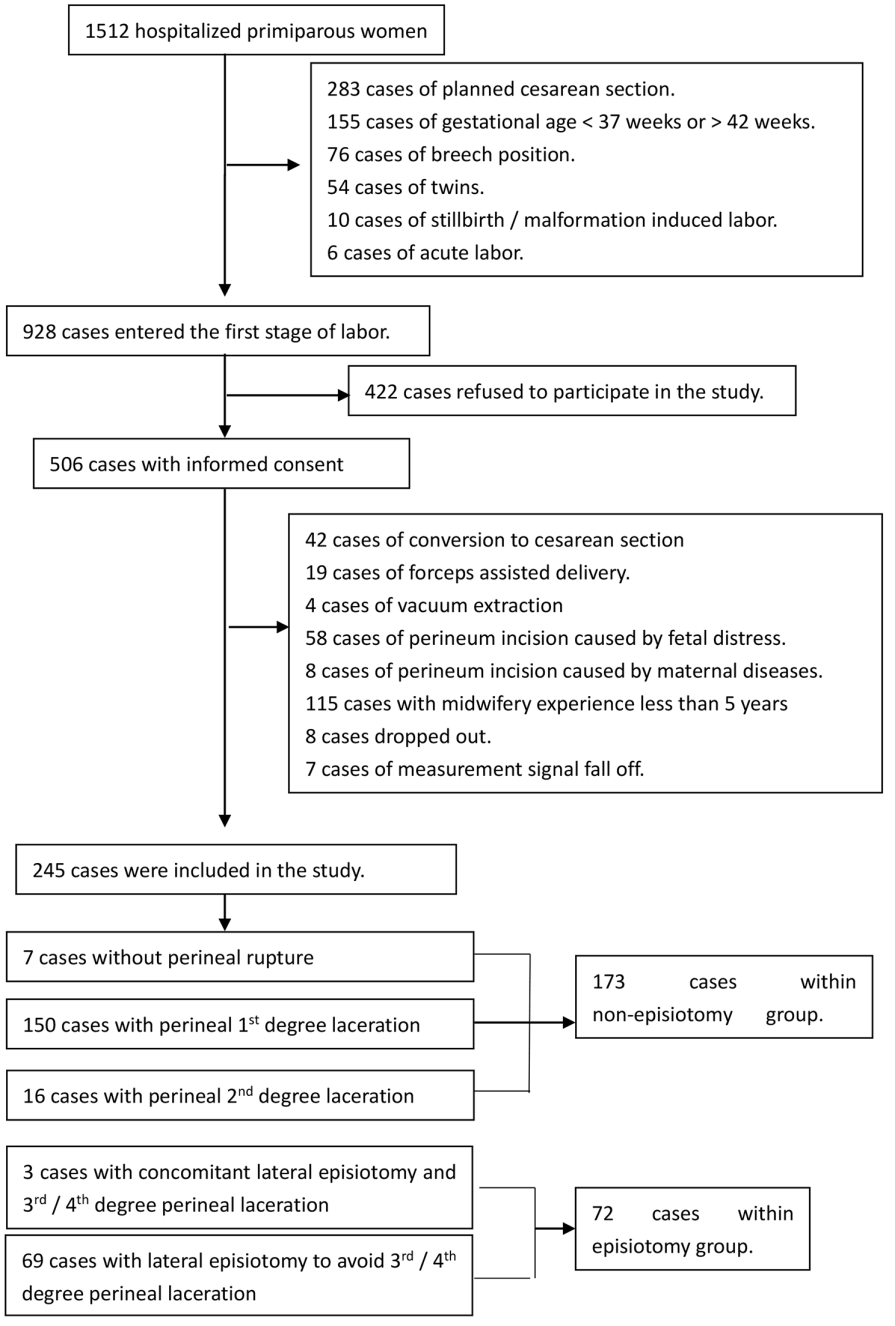
Study design and selection process for study participants

This study included a convenience sample of nulliparous women with a planned vaginal delivery in Ningbo Women & Children’s Hospital. The hospital ethics committee formally approved this study before study start (batch No.EC2020-059). The inclusion criteria were nulliparous women, between week 37 and 42, with a live, singleton fetus in cephalic presentation. The exclusion criteria were mental or neurological illness, poor communication by the mother, fetal malformations, midwifery experience < 5 years, cesarean or instrumental vaginal delivery, and episiotomy performed to avoid fetal distress. Informed consent was obtained from all women meeting the criteria prior to study commencement. During this study, 1513 nulliparous women were admitted to our hospital from December 2020 to March 2021. In total, 245 women were successfully enrolled for perineal stress investigation. The selection process is illustrated in Fig. 1. After delivery, participating women were grouped according to received treatment into non-episiotomy group (n = 173) and episiotomy group (n = 72).

### Selection of confounding factors

Based on the management guidelines and decision-making prediction models for the prevention of perineal laceration [11, 12], the following search terms were used: ‘perineal laceration’, ‘perineal injury’, ‘perineum tearing’, ‘laceration of perineum’, ‘episiotomy’, ‘obstetric anal sphincter injury’, ‘influencing factors’, ‘prediction’. A systematic search was conducted through CNKI, Wanfang data, Pubmed, Ebsco, Springer and other databases, and 18 influencing factors were listed. Five midwifery experts in China were selected to review the draft of influencing factors. The perineal length, perineal elasticity, perineal color, perineal thickness and perineal edema, which were five influencing factors that could not be dynamically measured and objectively evaluated in clinical practice - were excluded, and perineal stress was added. Inclusion and exclusion criteria excluded 8 factors, including gestational age, parity, uterine contraction, vaginal instrument delivery, drug-induced labor, perineal and vaginal laceration before delivery, fetal position and midwife’s work experience. Six influencing factors were finally included: maternal age, maternal BMI, presence of analgesia, duration of second stage, estimated birthweight, perineal stress.

### Evaluation of perineal stress and injuries

In this study, midwifery and delivery protocols were standardized. All participants received bilateral pudendal nerve block and were also given the option of epidural anesthesia. During the second stage of labor, the woman was placed in lithotomy position, with the midwife positioned on the woman’s right side, once the fetal head dropped to 3 cm below the ischial spine plane. When the fetal head was crowning, the woman was advised to push gently in order to avoid rapid delivery of the fetal head. Manual support was applied to slow down delivery of the fetal head and if required, the midwife’s fingers were also placed on the posterior perineum during crowning to relieve stress on the central perineum to prevent imminent anal sphincter injury. Women were not provided with perineal hot compresses or perineal massage.

**Fig. 2:**
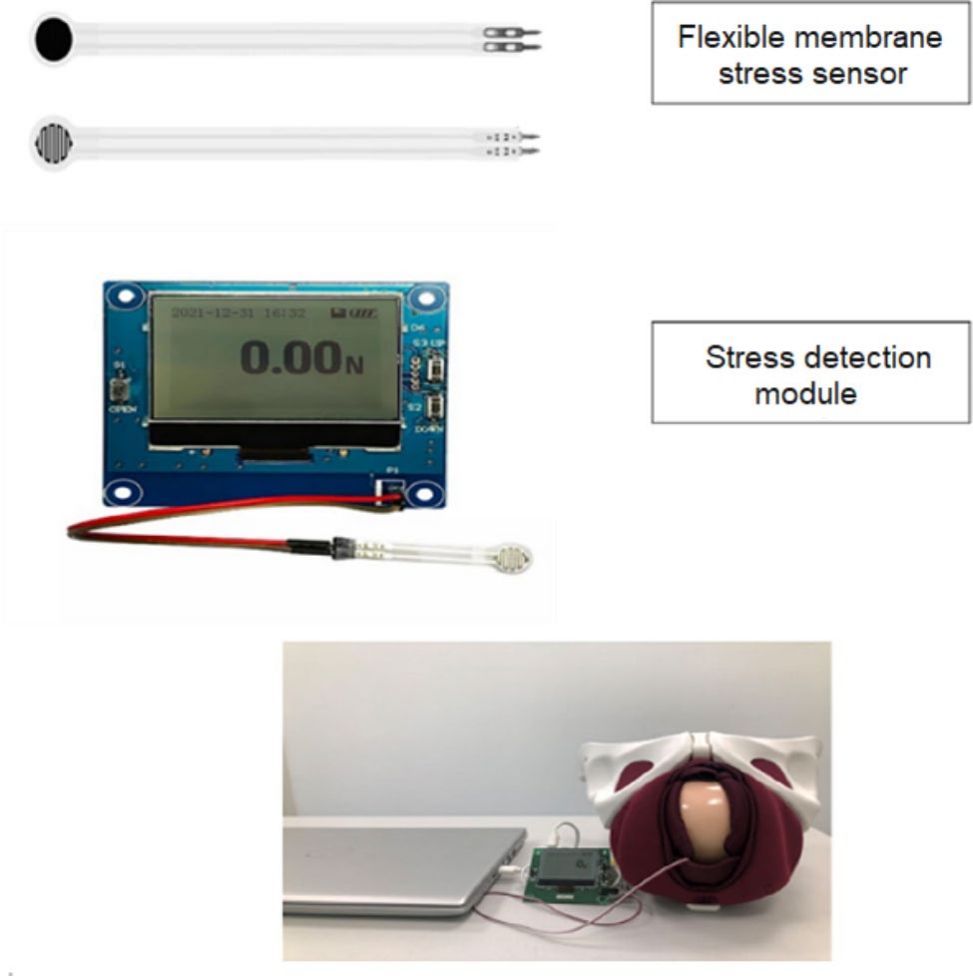
Essential setup for perineal stress monitoring and analyses

During intervals between contractions, following cleansing of the vaginal walls, the flexible membrane stress sensor (thickness < 0.3 mm, length 15 cm, range 1.5–200 N, response time < 1 ms) was placed by the attending midwife between the fetal head and vaginal wall above the anal fissure. The tail faced the right side of the woman to avoid interference with any episiotomy incision on the left side of the perineum. The sensor was fixed with a self-adhesive silicone dressing (2.5 cm x 2.5 cm). Once the entire fetal head pressed on the sensor, real-time perineal stress was measured through a stress detection module, and results were transmitted to a computer at a frequency of 100 ms/event. After delivery, stress values were noted, and the peak value recorded for each study participant. The attending midwife was not allowed to read the perineal stress measurements during delivery. The platform utilized for perineal stress monitoring and analyses is illustrated in Fig. 2.

The flexible stress sensor used in this study was based on a novel nano stress-sensitive material and an ultra-thin film substrate with a comfortable Young’s modulus. The platform was waterproof and had high stress sensitivity. Once the sensor sensed external stress, the resistance value of the sensor changed. Using a simple circuit, the sensor could convert stress signals into electrical signals, depending on stress intensity. The unit displayed stress values in Newton. This platform was uniformly calibrated prior to any readings.

Before the formal study, 50 primiparas with vaginal delivery were predicted through bivariate analysis, including 38 cases with no perineal rupture, perineal I degree and perineal II degree laceration, while the perineal stress was 101.92 ± 14.90 N; 12 cases underwent lateral episiotomy, the perineal stress was 137.79 ± 15.48 N. No perineal grade III and perineal grade IV cases were observed. The t-test value of perineal stress for primipara with differing perineal outcomes was 7.20, p < 0.05, which was statistically significant.

The degree of perineal laceration was assessed by visual inspection and/or palpation. However, when these techniques were not adequate, ultrasound used to assess potential perineal lacerations. Participants were divided into two groups, based on the requirement for episiotomy, as follows:

Episiotomy group (n = 72): lateral episiotomy to avoid 3rd degree perineal laceration.

Non-episiotomy group (n = 173): puerpera with no perineal rupture and 1st -2nd degree perineal lacerations.

### Statistical collection and analysis

Prior to delivery, the midwife was informed regarding maternal age, gestational age, body mass index before delivery, analgesia use, second stage commencement time, and estimated birthweight (calculated according to fetal biparietal diameter, head circumference, abdominal circumference and femur length measured by B-ultrasound). After delivery, the duration of the second stage, degree of perineal tear, and whether or not episiotomy was performed, were obtained from the electronic medical record and perineal stress measurement.

For continuous variables, we calculated mean and median values, standard deviation, and range. Logistic regression analysis was used to evaluate the correlation between perineal stress and episiotomy, with adjustment for maternal age, gestational age, BMI, duration of second stage of labor, analgesia, and estimated birthweight. This study described datasets using mean, standard deviation, median, minimum, and maximum values. T-test and chi-square test were used to describe the differences in each factor between the two groups. Logistic regression analysis was used to evaluate the correlation between perineal stress and episiotomy, post-adjusting for maternal age, gestational age, Maternal BMI, duration of second stage of labor, analgesia and estimated birthweight. The area under the curve (AUC) of receiver operating characteristic (ROC) was used to evaluate and define the threshold cut-off value for perineal stress, and whether to perform lateral episiotomy or not. The sensitivity, specificity, positive predictive value, negative predictive value, and accuracy of perineal stress at optimal cut-off value were calculated. SPSS 24.0® was employed for all statistical analyses.

This study explored the predictive value of perineal stress for episiotomy, with the area under the ROC curve (AUC) as the primary outcome measure. According to previous literature, AUC of 0.80 is considered to be clinically significant, and AUC = 0.70 is taken as the test benchmark [13]. we assumed that the proportion of non-episiotomy to episiotomy is approximately 2, α = 0.05 (unilateral), power = 0.80, while sample size of episiotomy is 63 cases and that of non-episiotomy is 126 cases, calculated by PASS 2021 software, totaling 189 cases. To account for a 10% drop-out rate, a minimum of 210 subjects should be included at baseline. Finally, 245 samples were included in this study.

## Results

### Participant characteristics

General characteristics for both study group participants are described in Table 1. There were no variations in maternal age, analgesic delivery and Maternal BMI between the two groups. However, participants that underwent episiotomy had a significantly prolonged second stage labor duration, increased estimated birthweight and perineal stress in comparison to non-episiotomy group

**Table 1:** General characteristics, labor stage details, and prevalence of analgesic delivery within study population

Index	Non-episiotomy (n = 173)	episiotomy (n = 72)	P Value
Maternal age (year)			
mean value ± standard deviation	27.58 ± 3.57	27.24 ± 3.56	0.495
median	28	27	
range	17~38	18~36	
Maternal BMI [Number (percentage)]			
≤ 24 Kg/m^2^	47(27.2)	14(19.4)	0.203
> 24 Kg/m^2^	126(72.8)	58(80.6)	
Duration of second stage of labor (min)			
mean value ± standard deviation	48.84 ± 25.63	68.92 ± 29.03	0.001*
median	43.00	67.50	
range	6~140	18~130	
Estimated birthweight [Number (percentage)]			
≤ 3500 g	131(76.2)	43(59.7)	0.012*
> 3500 g	42(23.8)	29(40.3)	
Perineal stress (Newton)			
mean value ± standard deviation	118.37 ± 19.21	140.50 ± 16.03	0.001*
median	119.05	140.59	
range	82.11~165.62	109.48~183.25	
Analgesic delivery (epidural) [Number (percentage)]			0.277
yes Number of cases(%)	153 (88.4)	67 (93.1)	
no Number of cases(%)	20 (11.6)	5 (6.9)	

*p < 0.05 Vs. non-episiotomy group participants

### Distribution of perineal stress across both study groups

**Fig. 3:**
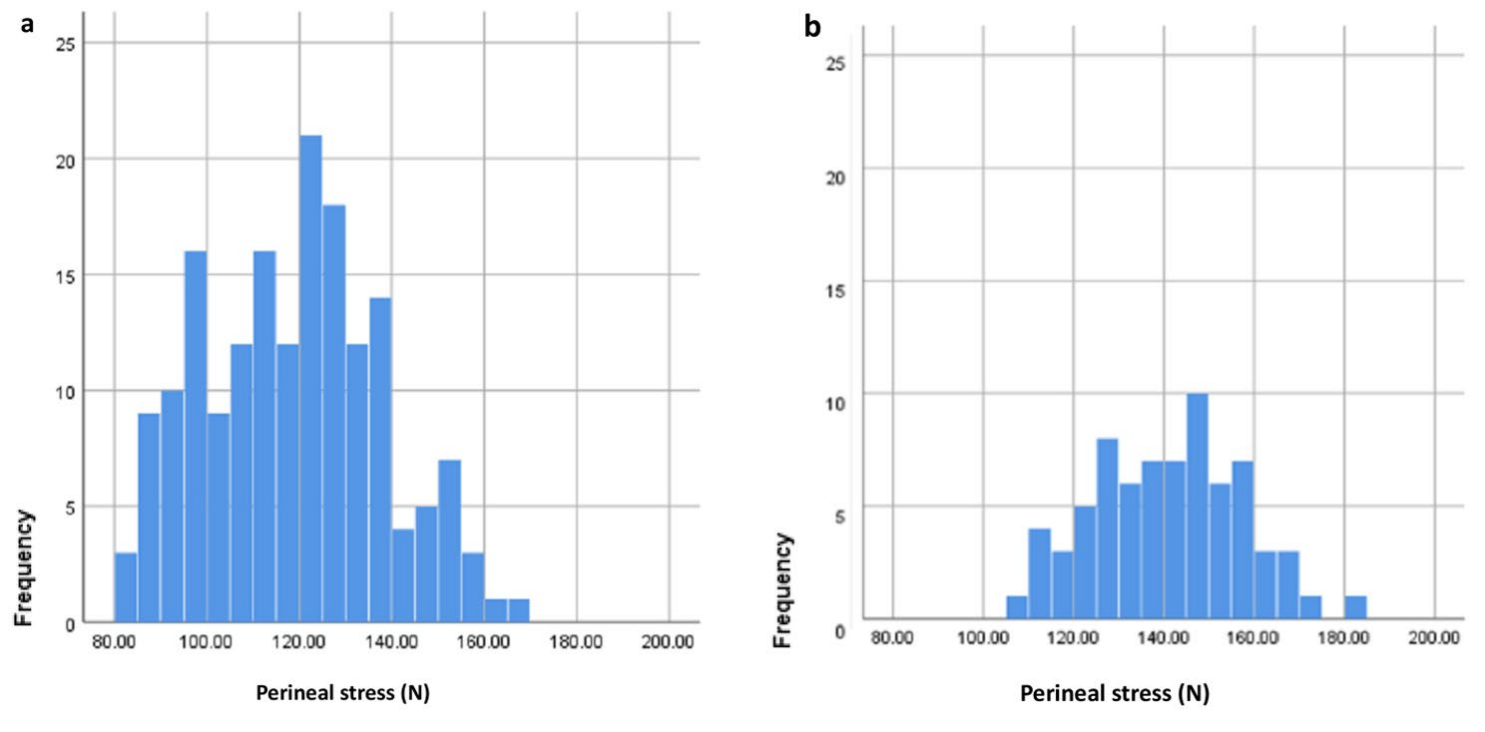
Histogram highlighting variations in perineal stress within study participants. **a**: non-episiotomy group **b**: episiotomy group

As shown in Fig. 3a and b, perineal stress of non-episiotomy group participants ranged between 82.11 and 165.62 N, while for episiotomy group participants this value ranged between 109.48 and 183.25 N.

### Logistic regression analysis of episiotomy-related factors

Logistic regression analysis was performed with all the related factors as independent variables, and whether the parturient underwent episiotomy as dependent variable. Continuous variables included age and perineal stress. The categorical variables were assigned the following values: Epidural analgesia: 0 = unused, 1 = used; Maternal BMI: 0 = less than or equal to 24 Kg/m2, 1 = greater than 24 Kg/m2. Estimated birthweights: 0 = less than or equal to 3500 g, 1 = greater than 3500 g. Episiotomy: 0 = no episiotomy, 1 = episiotomy. After adjustment for confounding factors, the statistical significance OR was 1.07 (95% CI 1.05–1.09). Such results indicated that perineal stress is a risk factor for episiotomy and is independent of the confounding factors highlighted in Table 2.

**Table 2:** The associations of perineal stress with episiotomy after adjustment for confounding factors

Factor	B	S.E.	Wald	df	Sig.	OR	95%CI (OR)	
							Lower	Upper
constant	-10.05	2.08	23.34	1.00	0.00	0.00		
Age(year)	-0.03	0.05	0.46	1.00	0.50	0.97	0.88	1.06
Maternal BMI	0.25	0.42	0.35	1.00	0.56	1.28	0.57	2.88
Duration of second stage of labor (min)	0.03	0.01	15.55	1.00	0.00	1.03	1.01	1.04
Analgesic delivery (epidural)	-0.18	0.63	0.08	1.00	0.78	0.84	0.24	2.89
Estimated birthweight	-0.13	0.37	0.12	1.00	0.73	0.88	0.42	1.83
Stress(Newton)	0.07	0.01	37.96	1.00	0.00	1.07	1.05	1.09

### Perineal stress values for episiotomy

**Fig. 4:**
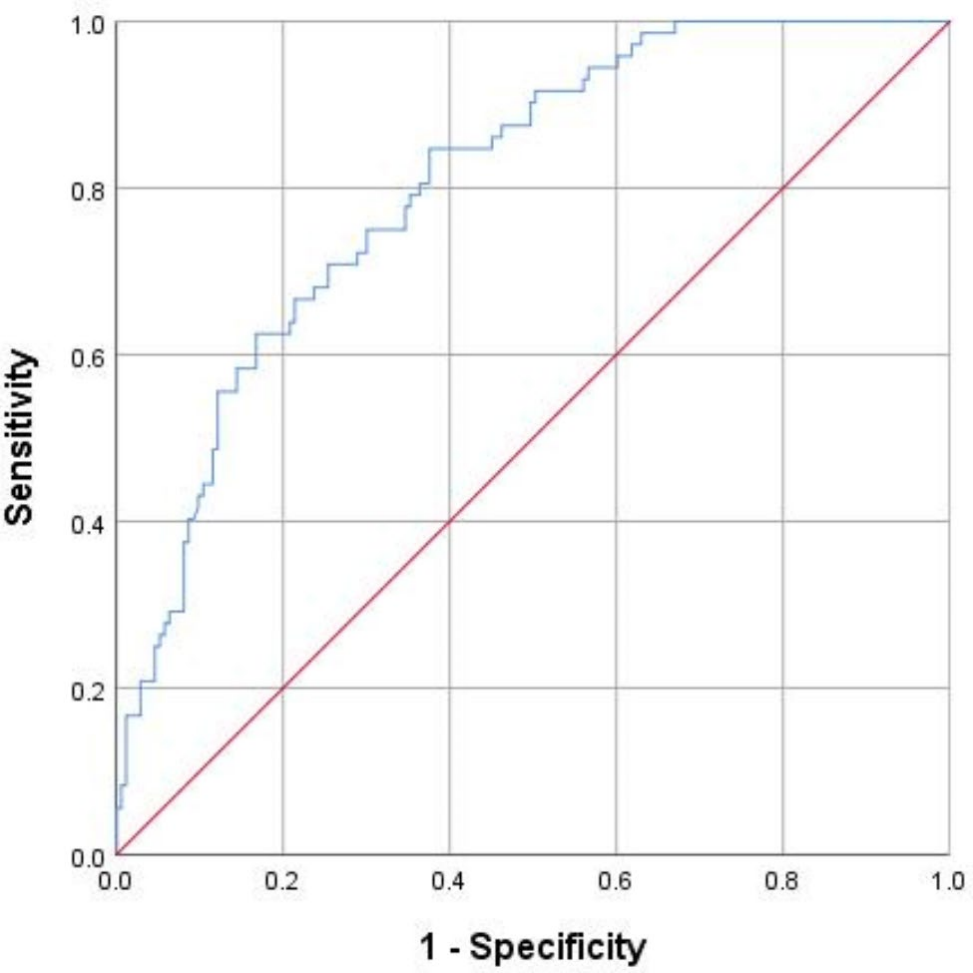
ROC curve illustrating the importance of perineal stress in performing episiotomy

According to the ROC curve analysis, the AUC of perineal stress for predicting episiotomy was 0.81 (95% CI 0.75–0.86) (Fig. 4). This indicated that the level of perineal stress has a high value in predicting episiotomy. Using the Youden index, perineal stress of 124.49 N had a sensitivity of 84.70%, specificity of 62.40%, a positive predictive value of 47.90%, and a negative predictive value of 90.9%.

### Validation of the predictive value of perineal stress

According to the optimized cut-off value of 124.49 N for perineal stress, participants were sub-divided into two categories, low stress category and high stress category, with the value of episiotomy analyzed accordingly. Predicted episiotomy based on stress cut-off compared to actual episiotomy, the negative predictive rate was 90.75%, the positive predictive rate was 48.41% (Table 3); this investigation revealed that the influence of stress upon episiotomy was statistically significant, with an OR value of 9.21 (95% CI 4.52–18.77) (Table 4).

After adjustment for potential confounding factors, the influence of perineal stress category upon the episiotomy was still statistically significant, with an OR value of 8.56 (95% CI 3.99–18.35). These results indicated that elevated perineal stress is a risk factor for episiotomy, and this positive association is independent of confounding factors (Table 5).

**Table 3:** Comparison of episiotomy rates between different stress categories

stress category	episiotomy		total	Predictive rate (%)
	no	yes		
low	108	11	119	90.75^a^
high	65	61	126	48.41^b^
total	173	72	245	

**a**: the negative predictive rate; **b**: the positive predictive rate

**Table 4:** The effects perineal stress category on episiotomy

Factor	B	S.E.	Wald	df	Sig.	OR	95%CI (OR)	
							Lower	Upper
stress category	2.22	0.36	37.38	1	0.00	9.21	4.52	18.78

**Table 5:** The associations of perineal stress category with episiotomy, post-adjusting for confounding factors

Factor	B	S.E.	Wald	df	Sig.	OR	95%CI (OR)	
							Lower	Upper
Constant	-3.23	1.49	4.67	1.00	0.03	0.04		
Age (year)	-0.03	0.05	0.30	1.00	0.59	0.98	0.89	1.07
Maternal BMI	0.18	0.41	0.19	1.00	0.66	1.20	0.54	2.65
Duration of second stage of labor (min)	0.03	0.01	16.46	1.00	0.00	1.03	1.01	1.04
Estimated birthweight	0.07	0.36	0.03	1.00	0.85	0.94	0.47	1.89
Analgesic delivery (epidural)	0.08	0.62	0.02	1.00	0.90	1.08	0.32	3.67
stress category	2.15	0.39	30.45	1.00	0.00	8.56	3.99	18.35

## Discussion

The salient findings of this study are as follows: (1) a positive association exists between perineal stress and episiotomy during vaginal delivery; (2) Participants with elevated perineal stress often led the midwife to implement an episiotomy; (3) The association between perineal stress and episiotomy is independent of maternal age and BMI, estimated birthweight, duration of the second stage of labor, and presence of analgesia; (4) A perineal stress cut-off value of 124.49 N can be helpful to predict midwives’ decision on episiotomy.

Several randomized controlled trials highlighted that lateral episiotomy would be adept for lowering the risk of OASIS manifestation [14], although when opting for lateral episiotomy, clinicians were careful to balance OASIS risk against the possibility of morbidity issues by using this procedure [15]. Within Chinese midwifery concepts, when “severe perineal tear is inevitable” and “perineal tightness” is present, perineal incision is usually preferred to avoid anal sphincter injury [16]. In order to prevent midwives from misjudging the occurrence of anal sphincter injury [17], Rao Lin [10] and Guo Lin [11] attempted to establish the evaluation and prediction model of influencing factors for episiotomy, and believed that perineum length, elasticity, degree of edema in perineum, prenatal vaginal laceration, estimated birthweight, degree of maternal cooperation, uterine contraction, and midwife’s years of delivery affected the decision-making of Chinese midwives in implementing episiotomy. However, in clinical practice, midwife interviews suggest that prediction is difficult to achieve, due to the excessive / variable evaluation items and short evaluation time [18]. Midwives require more simple and objective indicators to provide reference for clinical decision-making [19].

During normal vaginal delivery, the perineum is compressed as the fetal head descends. This can induce perineal stretching by up to 170% in the transverse direction and 40% in the vertical direction during crowning [20]. However, the level of three-dimensional deformation of the anal sphincter, due to stress overload during delivery, is poorly characterized [21]. Consequently, stress measurements of actual perineal stretching during delivery can be of great assistance to indicate requirement for additional therapeutic interventions. However, presently, technical tools to analyze shifts in perineal tissue tension during delivery remain lacking within routine clinical settings [22]. The criteria involved when a selective episiotomy is indicated are far from consistent, and this issue requires additional effort by scientific societies towards a more clearly defined, standardized description, with a definitive protocol guideline [23]. Consequently, this study assessed stress-sensing technology, which is also non-invasive, and convenient to dynamically monitor perineal stress once the fetal head drops during vaginal delivery.

The area under the ROC curve (AUC) for perineal stress, as a means of predicting episiotomy, was 0.81 (95% CI: 0.75–0.86), indicating that perineal stress had a clinical predictive value for episiotomy [13]. At the Youden index, perineal stress was 124.49 N, sensitivity was 84.70%, specificity was 62.40%, positive predictive value was 47.90%, while negative predictive value was 90.9%. Through employment of elevated sensitivity and negative predictive value alone, our study finding can consequently provide a reference value for junior midwives to avoid unnecessary episiotomies when perineal stress is lower than 124.49 N. The positive predictive value was 47.90%, which may be due to the fact that only the maximum stress value was measured in this study without considering the stress duration, thereby reduced the positive predictive effect. Nevertheless, once perineal stress exceeding a value of 124.49 N is record, although this stress is not equivalent to perineal stress in the actual occurrence of OASIS, midwives should be aware of this, and prepare for performing an episiotomy, or ask senior midwives and/or doctors for assistance.

Midwife clinical expertise has been found to be a critical component in decision-making regarding episiotomy [24]. In our study, each midwife had standardized training and certification and had delivered over 600 newborns each. The perineum incision rate of primipara in this study cohort was 29.38%, far lower than the national mean of 41.70% [25]. This study hopes to provide reference for several areas with high episiotomy rates and for midwives lacking in sufficient delivery experience - through quantitative data or by providing training assistance for midwifery interns.

The method described was not able to measure perineal tissue tension, though measured a force acting upon the perineum during fetal head expulsion. Cohort size was also limited, warranting additional studies using large cohort sizes to validate and extend our findings. Confounding factors, such as maternal pushing and use of perineal protection, could have partly compromised data validity. The study was conducted at a single center, whereby multi-centers studies would be useful for clinical validation. Future studies should ideally incorporate women with differing age brackets and ethnic backgrounds, in order to expand our findings. Due to funding constraints and a limited sample size, this study only found three women with OASIS within the study cohort. However, in the future, we plan to conduct a study focusing on the ability of perineal stress measurement to predict OASIS and other severe pelvic floor injuries.

## Conclusion

This study – to the best of our knowledge – pioneered the concept of monitoring perineal stress in nulliparous women during vaginal delivery and was successful in probing its clinical value for indicating the use of episiotomy during vaginal delivery. Our study finding can consequently provide a reference value for junior midwives to avoid unnecessary episiotomies under 124.49 N. In addition, midwives should be aware of the risk of episiotomy, or ask senior midwives and/or doctors for assistance, once perineal stress exceeding a value of 124.49 N is recorded.

## Data Availability

The datasets generated and/or analyzed during the current study are available from the corresponding author upon reasonable request.

## Abbreviations

OASIS: obstetric anal sphincter injuries.
